# Cerebral Blood Flow Links Insulin Resistance and Baroreflex Sensitivity

**DOI:** 10.1371/journal.pone.0083288

**Published:** 2013-12-16

**Authors:** John P. Ryan, Lei K. Sheu, Timothy D. Verstynen, Ikechukwu C. Onyewuenyi, Peter J. Gianaros

**Affiliations:** 1 Department of Psychiatry, University of Pittsburgh, Pittsburgh, Pennsylvania, United States of America; 2 Department of Psychology, University of Pittsburgh, Pittsburgh, Pennsylvania, United States of America; 3 Department of Psychology, Carnegie Mellon University, Pittsburgh, Pennsylvania, United States of America; University of Sao Paulo, Brazil

## Abstract

Insulin resistance confers risk for diabetes mellitus and associates with a reduced capacity of the arterial baroreflex to regulate blood pressure. Importantly, several brain regions that comprise the central autonomic network, which controls the baroreflex, are also sensitive to the neuromodulatory effects of insulin. However, it is unknown whether peripheral insulin resistance relates to activity within central autonomic network regions, which may in turn relate to reduced baroreflex regulation. Accordingly, we tested whether resting cerebral blood flow within central autonomic regions statistically mediated the relationship between insulin resistance and an indirect indicator of baroreflex regulation; namely, baroreflex sensitivity. Subjects were 92 community-dwelling adults free of confounding medical illnesses (48 men, 30-50 years old) who completed protocols to assess fasting insulin and glucose levels, resting baroreflex sensitivity, and resting cerebral blood flow. Baroreflex sensitivity was quantified by measuring the magnitude of spontaneous and sequential associations between beat-by-beat systolic blood pressure and heart rate changes. Individuals with greater insulin resistance, as measured by the homeostatic model assessment, exhibited reduced baroreflex sensitivity (*b* = -0.16, *p* < .05). Moreover, the relationship between insulin resistance and baroreflex sensitivity was statistically mediated by cerebral blood flow in central autonomic regions, including the insula and cingulate cortex (*mediation coefficients* < -0.06*, p-values* < .01). Activity within the central autonomic network may link insulin resistance to reduced baroreflex sensitivity. Our observations may help to characterize the neural pathways by which insulin resistance, and possibly diabetes mellitus, relates to adverse cardiovascular outcomes.

## Introduction

The central nervous system monitors peripheral energy balance via hormones that are actively transported across the blood brain barrier [1–3]. Insulin, a hormone excreted by the pancreas in response to increased blood glucose levels, has receptors that are widely distributed throughout the brain [4]. Administration of insulin into the brain has a diverse range of effects, including effects on food intake[5], cognitive performance[6], autonomic outflow [7–9], and peripheral infusions of insulin increase the ability of the arterial baroreflex to alter peripheral sympathetic activity in response to changes in blood pressure [10]. Insulin resistance (IR) is a pathological state in which the effects of insulin on peripheral tissues are reduced, primarily as a result of overeating, obesity and lack of physical activity. As IR develops, the transport of insulin across the blood brain barrier is reduced, thereby altering its ability to affect the central nervous system [4,11]. Although it is established that IR is associated with altered autonomic activity, primarily due to increased sympathetic outflow [12,13], no studies have examined activity in the human brain that may account for this relationship.

A distributed network of brain regions play a role in regulating the arterial baroreflex [14–16]. Within this network, brainstem cell groups participate in the rapid and homeostatic tuning of baroreflex sensitivity (BRS) to adjust heart rate, cardiac contractility, and vascular resistance in response to blood pressure oscillations via the autonomic nervous system [17–19]. Hence, baroreceptor signaling is relayed via the afferent limbs of the vagal and glossopharyngeal cranial nerves to the nucleus tractus solitarius, which in turn projects to the rostral and caudal territories of the ventrolateral medulla and nucleus ambiguus. These projections enable a homeostatic decrease in efferent sympathetic cardiac and vascular outflow that is coupled with increased parasympathetic cardiac outflow in response to transient blood pressure elevations [14,16,20]. Although brainstem cell groups comprise a critical part of the baroreflex arc, supramedullary areas within the forebrain are able to exert modulatory influences over the baroreflex via their reciprocal projections with preautonomic cell groups within the brainstem. These regions include the hypothalamus [21], amygdala [22], sensorimotor cortex [23], anterior cingulate cortex, and insula [24,25]. Collectively, these regions comprise core components of the so-called central autonomic network (CAN)[26], which has been consistently linked to peripheral measures of autonomic function in humans [27].

To date, no human research has tested whether alterations in the CAN may link aspects of autonomic dysregulation, such as altered baroreflex control, with alterations of circulating insulin. Such a link is plausible insofar as several regions of the CAN overlap with those that are also (a) sensitive to the effects of insulin and (b) display altered activity in the context of IR, which itself has been linked to reduced baroreflex control [28–30] and adverse cardiovascular health outcomes [31]. 

In addition to their capacity for the efferent modulation of the baroreflex, regions of the CAN are also involved in the afferent monitoring of baroreceptor signaling [32,33]. In this regard, it is noteworthy that IR is associated with cardiovascular and autonomic alterations, including reduced arterial compliance [34] and damage to vagal nerve fibers [35,36]. Hence, IR-related alterations in peripheral BRS could also relate to changes in activity within the CAN via afferent mechanisms.

Although a growing animal literature is documenting the brain circuits that may link IR and baroreflex function, no studies have examined the central nervous system pathways that may statistically mediate such a link in humans. To test the hypothesis that activity in the CAN could partially account for the relationship between IR and BRS, we measured resting cerebral blood flow (rCBF) using arterial spin-labeling perfusion MRI and identified regions of the brain that statistically mediated the relationship between IR and BRS. We hypothesized that activity in regions within the CAN, as measured by rCBF, would show associations with individual differences in IR. Furthermore, we hypothesized that the activity in these regions would be associated with spontaneous BRS. Finally, a statistical mediation analysis was conducted to identify brain regions that statistically accounted for any observed relationships between IR and BRS.

## Methods

### Ethics Statement

This research was approved by the University of Pittsburgh Institutional Review Board. Participants provided written informed consent prior to participating in the study.

### Participants

Participants were recruited by mass mailings to Allegheny County, Pennsylvania, USA. Exclusion criteria included (i) prior cardiovascular surgery (including coronary bypass, carotid artery, or peripheral vascular surgery); (ii) self-reported history of cardiovascular disease (including treatment for or diagnoses of hypertension, stroke, myocardial infarction, congestive heart failure, and atrial or ventricular arrhythmias); (iii) self-reported current or past diagnoses of a substance abuse or mood disorder as confirmed on interview using the Patient Health Questionnaire [37]; (iv) chronic kidney or liver conditions, diagnosed type 1 or 2 diabetes, or any pulmonary or respiratory disease; (v) prior cerebrovascular trauma involving loss of consciousness; (vi) prior neurosurgery or any neurological condition; (vii) taking psychotropic, lipid lowering, or cardiovascular medications; (viii) having claustrophobia or metallic implants; or (ix) pregnancy (verified by urine test in females). The University of Pittsburgh Institutional Review Board approved all study procedures and informed consent was obtained from all participants. 

One hundred fifty-five participants completed the study between 2008 and 2011. Five participants did not have blood data, and 45 participants did not have fasting insulin values that were detectable. Participants with missing data were excluded from analyses. Participants with undetectable insulin levels had lower waist circumference (t (153) = 3.48, p < .01), fasting glucose (t (150) = 2.16, *p* < .05), triglycerides (t (150) = 3.53, *p* < .01) and higher HDL (t (150) = 2.74, *p* < .01). There were no differences in age, sex or LDL between groups (all *p* > .20). Additionally, eleven participants were excluded due to poor MRI quality or excessive movement. Two participants had extreme BRS values (3SD from the mean) and were excluded as outliers. Thus, 92 participants had complete data and were included in analyses. Participant characteristics are displayed in Table 1.

**Table 1:** Participant characteristics (n = 92).

Variable	Value
Age, yr	40.6 (6.4)
Sex (M/F), n (%)	48 (52)/44(48)
Waist Circumference (in)	36.95 (4.8)
Insulin (μU/mL)	8.29 (5.9)
Glucose (mg/dL)	90.34 (15.0)
Systolic Blood Pressure	126.55 (11.3)
Baroreflex Sensitivity (ln msec/mmHg)	2.13 (0.4)

M = male; F = female

Data are presented as mean (standard deviation) unless otherwise noted.

### Procedure

Participants completed two separate study protocols (median intersession interval = 7 days, with MRI testing occurring first across all subjects). Participants were instructed to abstain from food, drink, exercise, caffeine and tobacco products for 8 h and alcoholic beverages for 12 h prior to both sessions. The first session included a neuroimaging scan that included a resting arterial spin-labeling perfusion protocol (described below). The second session included continuous blood pressure monitoring (described below) while in a plastic MRI scanner replica. 

BRS was quantified using the Finometer® system. The Finometer uses a photoplethysmograph to detect, digitally sample and store a continuous measure of estimated brachial pressure. Finger arterial blood pressure first calibrated to brachial blood pressure using an upper-arm cuff and corrected to account for the height difference between the finger and heart [38]. Data were visually inspected offline prior to analysis, described below.

BRS was computed using the *x*BRS software package, which uses a crosscorrelation, time-domain method to quantify “spontaneous” associations between systolic blood pressure and interbeat interval values [39,40]. BRS was computed by cross-correlating systolic blood pressure and interbeat interval in 10-s epochs using a delay ranging from 0 to 5 s and providing up to six correlations for each delay and epoch. For each epoch, the delay yielding (i) the highest correlation and (ii) a coefficient of determination significant at *p* < 0.01 was retained, and the respective regression slope was taken as the BRS estimate in msec/mmHg. If these conditions were unmet, a BRS estimate was not calculated. Resting BRS estimates were averaged over the last 5 minutes of the 8 minute baseline. As expected, BRS values were skewed and were subjected to log-normal transformation.

Following an overnight fast, blood was drawn just prior to MRI scanning. Serum was analyzed using a Synchron CX chemistry analyzer (Beckman-Coulter, Brea, CA) using reagents for glucose, triglyceride, HDL and total cholesterol. LDL cholesterol values were estimated by subtracting the HDL cholesterol level from total cholesterol. Insulin was quantified using the Immulite Immunoassay System (Siemens). IR was computed using Homeostatic Model Assessment of IR (HOMA-IR) values, which approximates IR based on fasting glucose and insulin levels [41]. Prior to analyses, HOMA-IR values were natural log adjusted to reduce skew.

### Brain Imaging Acquisition

Neuroimaging data were acquired on a 3T Trio TIM whole body scanner (Siemens), equipped with a 12-channel, phased-array head coil. Resting perfusion images were acquired with a pulsed arterial spin-labeling sequence. For this sequence, interleaved perfusion images with and without arterial spin labeling were obtained over a 5-minute, 28-second period using gradient-echo echo-planar imaging. The pulsed arterial spin-labeling sequence used a modified flow-sensitive alternating inversion recovery method [42], specifically applying a saturation pulse 700 msec after an inversion pulse. To reduce transit artifact, a 1000-msec delay separated the end of the labeling pulse and the time of image acquisition. Resting perfusion image acquisition parameters were: field of view: 240×240 mm (64x64 matrix); repetition time: 4000 msec; echo time: 18 msec; and flip angle: 90°. Twenty-one slices (5 mm thick, 1 mm gap) were acquired sequentially in an inferior-to-superior direction for each brain image, yielding 80 total perfusion images (40 labeled and 40 unlabeled; 3 initial discarded images allowing for magnetic equilibration). Two additional unlabeled (control) perfusion images using the same parameters but a longer TR, 8000 msec were acquired as reference for the equilibrium brain tissue magnetization. Functional images were coregistered and normalized to Montreal Neurological Institute (MNI) space via a T1-weighted three-dimensional magnetization-prepared rapid gradient echo anatomical image (field of view: 256 x 208 mm (256x208 matrix); repetition time: 2100 msec; inversion time: 1100 msec; echo time: 3.29 msec; flip angle: 8 degrees, 192 slices; 1 mm thick, no gap).

### Brain Image Processing and Analysis

Data processing was completed using SPM8 (Wellcome Trust Centre for Neuroimaging; http://www.fil.ion.ucl.ac.uk/spm/software/spm8/). Resting arterial spin labeling perfusion and the reference images were realigned to the first image of the series by rigid body transformation and smoothed with a 12mm full-width-at-half-maximum isotropic Gaussian kernel. Pairwise difference images of label and control were calculated and then submitted to the Standard Kinetic Model, along with the mean reference images, to construct rCBF images [43]. This yielded 40 rCBF images for each participant, which were then averaged and normalized to MNI space for analysis. 

Mediation analyses were conducted using bootstrap regression analysis of voxelwise observations (BRAVO) software (https://sites.google.com/site/bravotoolbox/). When examining the relationship between two variables (in this case, IR and BRS), mediation analysis examines the amount of covariance that is accounted for by an intervening variable (i.e., cerebral blood flow)[44]. Accordingly, two multiple regressions were performed in mediation modeling: first the intervening variable (rCBF) was regressed on the independent variable (IR) to determine the effect of IR on rCBF (path a). The dependent variable (BRS) then was regressed on the intervening variable (rCBF) while controlling for the independent variable (IR) to determine the effect of rCBF on DV (path b). The mediation effect, or the indirect effect, of IR on BRS via rCBF was calculated by the product of the effects from the two regressions (path a*b). Bootstrapping is a statistical resampling method used to estimate a statistical parameter, in this case the mediation effect of the intervening variable [45], through repeated resampling of the data [46]. In the present study, we estimated whether rCBF in voxels accounted for a significant portion of the covariance between IR and BRS. Models were run with 2000 bootstrap iterations and included resting systolic blood pressure, sex, age, waist circumference and global rCBF as covariates. As a result, a statistical parameter map of rCBF mediation to the relationship of IR and BRS was constructed (i.e., p-value map), allowing for the examination of voxel-wise mediation effect. Once voxels were identified that mediated the IR and BRS Association, a cluster threshold was applied to maintain a whole brain threshold of *p* < .05. AlphaSim software (http://afni.nimh.nih.gov/afni/doc/manual/AlphaSim) was used to determine the appropriate correction threshold for the image mask (*p* = .005, voxel size = 3mm, FWHM x, y, z = 12.0, 12.5, 13.6, 5000 iterations, k = 109). 

Covariance of IR and BRS was examined by using hierarchical linear regression analyses conducted in SPSS (v. 20; IBM, Inc., Armonk, NY). Potential confounding variables (age, sex, systolic blood pressure, waist circumference) were entered in the first step and the variable of interest was entered in a second step.

## Results

### Relationship between IR and BRS

The hierarchical regression of BRS on IR showed that IR was predictive of BRS in the expected direction (*b* = -0.16, p < 0.05; Figure S1). The variance explained by IR was independent of sex, age, waist circumference and resting systolic blood pressure (*F*_*change*_ (1, 85) = 6.48, *p* < .05, *R* ^*2*^_*change*_ = 0.06). 

### Associations between IR and rCBF (Path a)

In a whole brain analysis, a large cluster encompassing several regions displayed positive associations between IR and rCBF (Figure S2, Table S2a). The cluster included the bilateral somatosensory cortex, left parietal operculum reaching into the insular cortex, left dorsolateral prefrontal cortex and bilateral posterior cingulate cortex (k = 643, *p* < .01). In the whole brain analysis using a corrected statistical significance threshold, no association was seen between IR and rCBF in other hypothesized components of the CAN, such as the amygdala, hypothalamus and perigenual anterior cingulate cortex. Likewise, no regions displayed inverse relationships between IR and rCBF.

### Associations between rCBF and BRS (Path b)

A number of regions showed negative associations between rCBF and BRS (*p*_*corrected*_ < .05, Figure S3, Table S2b). A substantial cluster of regions previously linked to baroreflex regulation was associated with BRS including the right amygdala and insula (k = 432, *p* < .001). Additional regions in the CAN that displayed inverse associations with BRS included left anterior cingulate cortex (k = 241, p < .001), right mid-cingulate cortex (k = 647, p < .001), bilateral posterior cingulate cortex (k = 134, p < .001) and a broad region encompassing the right dorsolateral prefrontal cortex, insula and operculum (k = 1794, p < .001). One cluster of activity in the right superior temporal gyrus displayed a positive association with BRS (*x, y, z*: 45, -52, 1; k = 151).

### Regions that statistically mediate IR and BRS associations (Path a*b)

**Figure 1:**
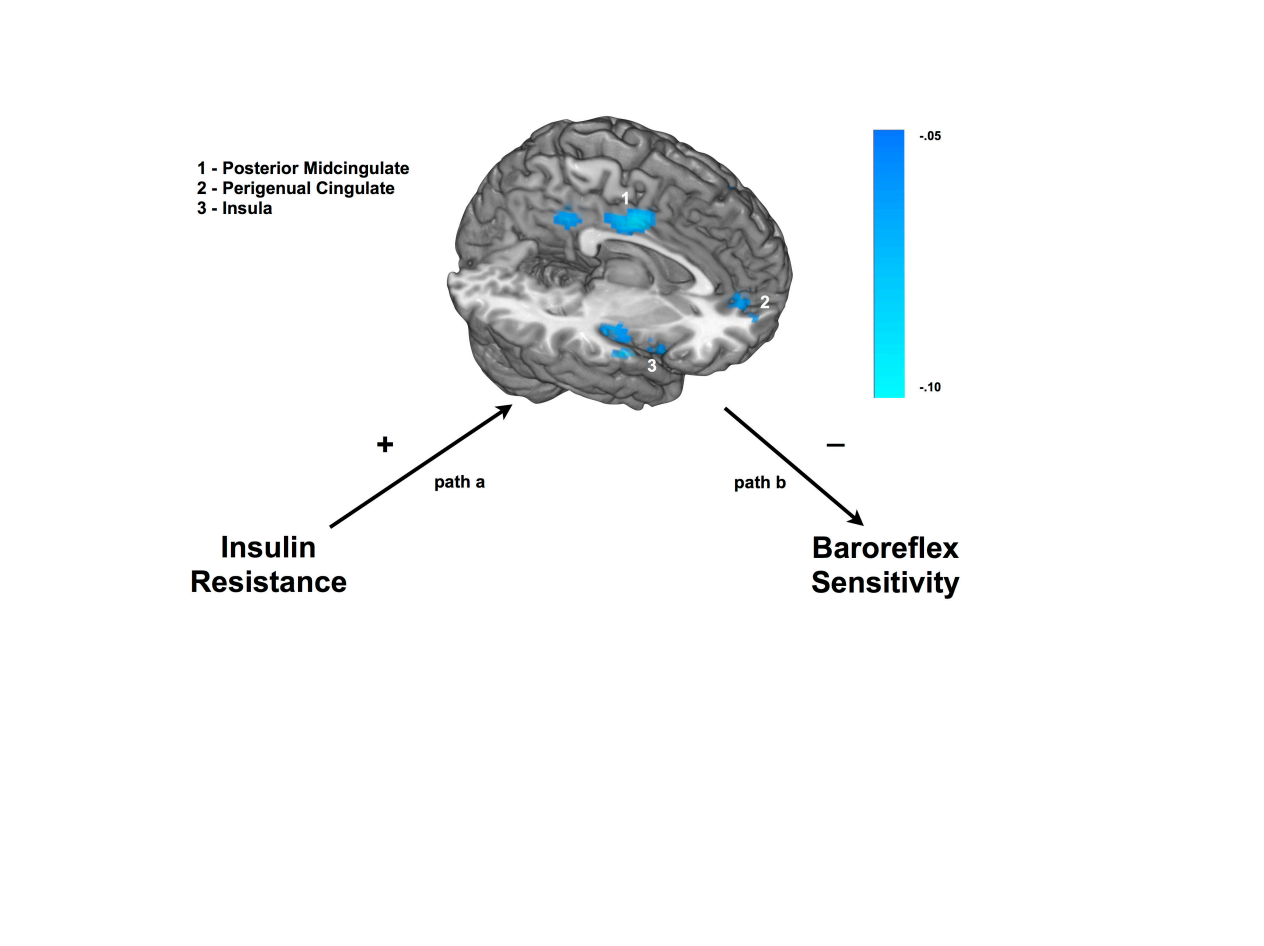
Regions that statistically mediated the relationship between insulin resistance (IR) and baroreflex sensitivity (BRS). Results presented are ‘path a*b’ regression coefficient output from the BRAVO mediation toolbox. Regions displayed statistically mediated the relationship between IR and BRS after covarying for resting systolic blood pressure, age, sex, waist circumference and global cerebral bloodflow. To correct for multiple comparisons, a cluster threshold was utilized (*p*-uncorrected = .005, k = 109) to maintain a whole brain threshold of *p* < .05.

rCBF in a distributed set of brain regions mediated the relationship between IR and BRS (*p*_*corrected*_ < .05, Figure 1, Table 2). As hypothesized, regions previously associated with baroreflex regulation, as well as regions that show alterations in activity in individuals with IR, statistically mediated the relationship between IR and BRS (*p*_*corrected*_ < .05). These regions included the right insula (k = 809, *p* < .001), as well as bilateral perigenual anterior cingulate (k = 176, *p* < .01). Additionally, bilateral regions of the dorsolateral prefrontal cortex reached threshold (k = 295, *p* < .001), as did a cluster in the posterior midcingulate cortex and posterior cingulate cortex (k = 429, *p* < .001). In all cases, values were negative reflecting a positive relationship between IR and rCBF, which in turn predicted a decrease in BRS. 

**Table 2:** Brain regions that statistically mediated the relationship between insulin resistance and baroreflex sensitivity.

Side	Region	Brodmann Area	MNI Coordinates			Number of voxels	Peak intensity
			x	y	z		
R	Insula, Dorsolateral Prefrontal Cortex	6, 4, 9, 13, 47, 22, 8, 3, 44, 38, 21, 46, 2, 42, 43, 1, 45	36	-1	55	809	-0.107
R, L	Perigenual Anterior Cingulate	10, 11, 24, 32	0	47	-2	176	-0.065
L	Postcentral Gyrus	4, 6, 21, 22, 40, 41, 42, 43, 41	-60	-16	13	126	-0.082
R, L	Posterior Midcingulate Cortex, Posterior Cingulate Cortex	6, 23, 24, 31, 32	3	-10	37	429	-0.092
L	Dorsolateral Prefrontal Cortex	2, 3, 4, 6, 8, 9	-27	32	46	295	-0.099

Greater insulin resistance (IR) was associated with increased resting cerebral blood flow (rCBF), which in turn was related to decreased baroreflex sensitivity (BRS). Next to each left (L) or right (R) side is an approximation of the brain region as well as the Brodmann Area if applicable. Montreal Neurological Institute (MNI) coordinates indicate the peak activation for each cluster: x = right (+) to left (-), y = anterior (+) to posterior (-), z = superior (+) to inferior (-). Clusters are derived from a whole brain analysis with an uncorrected height threshold of *p* < .005 and extent threshold of k = 109. Peak intensity values in the final column are for the voxel with the strongest indirect effect (a * b) and are derived from the probability maps generated from the BRAVO mediation toolbox. All regions displayed negative values reflecting the positive association between IR and rCBF, which in turn predicted decreased BRS.

## Discussion

Regional blood flow to brain regions involved in autonomic regulation statistically mediated the inverse relationship between IR and BRS. These regions included the perigenual anterior cingulate cortex and insula. As such, the present findings highlight the role of forebrain regions of the human brain in linking alterations in BRS that accompany IR. Additionally, a region encompassing the posterior midcingulate and posterior cingulate cortices statistically mediated between IR and BRS. Although these regions are generally not thought to be strongly or directly involved in the regulation and monitoring of autonomic activity [47], their functional connections with other regions which are part of the CAN may account for their statistical significance in the present analysis. Specifically, the posterior midcingulate cortex has been shown to have strong functional connectivity with the insula, whereas the posterior cingulate cortex is functionally connected to the perigenual anterior cingulate cortex [48]. A recent meta-analysis of the neural correlates of autonomic function suggested that the posterior cingulate cortex is part of a network associated with parasympathetic control [27]. This meta-analysis further indicated that markers of both sympathetic and parasympathetic autonomic activity are associated with activity in regions of the CAN, including the anterior cingulate and insular cortices. Finally, both the insula and midcingulate cortex were suggested to be associated with indicators of autonomic activity across affective, cognitive and somatosensory tasks, indicating their centrality in autonomic monitoring and modulation across diverse behavioral states. In aggregate, our findings would appear to agree with the main results of this meta-analysis. However, the current study did not examine functional or structural relationships between regions that are presumably involved in central autonomic regulation, leaving unclear how the interplay between autonomic control regions relates to baroreflex function in the context of insulin resistance. Accordingly, this will be an important direction for future research. 

A notable finding of the present study is the degree of overlap in between regions that mediate IR and BRS, and regions that have previously been linked to autonomic regulation. The perigenual cingulate cortex and insula are key nodes in the CAN and their activity has been correlated with changes in heart rate and blood pressure evoked through a variety of methods including the Valsalva maneuver [49], exercise [50], and cognitive tasks [15]. A growing literature is also showing that these regions are sensitive to the effects of insulin, and these effects may be moderated by individual differences in anthropometric measures of relative adiposity or obesity [51]. Notably, the present study controlled for waist circumference, an indirect indicator of adiposity, suggesting a unique relationship between IR and rCBF in cortical regions.

Regions such as the insula have been linked to functions that are involved in homeostatic control and food intake [52]. Previous research has implicated the insula as a key node for interoception [53], and there are extensive interconnections between the insula and cingulate cortex [54]. The present study found a lateralized effect, and the meta-analysis noted above suggested that right anterior insula might be particularly involved in sympathetic regulation. However, the studies within the meta-analysis included only measures of electrodermal activity as a marker of sympathetic activity. Future research will be needed to confirm any laterality effects and clarify the significance with regard to autonomic function.

The insula is also a key component of a network of regions involved in food reward [55] and exhibits altered activity in obese individuals [56]. Previous work from our group has demonstrated altered connectivity of the insular cortex in conjunction with IR [57]. Further research is needed to clarify whether the alterations in insular activity, in this case as measured by rCBF, are the result of a loss of insulin signaling, increased plasma insulin values that co-occur with IR, or are the result of perception of autonomic activity. Additionally, it will be important to extend this work to populations with diagnoses of type 2 diabetes to understand how these relationships may change as the body loses control of glucose regulation.

Notably, participants were free of diagnosed type 2 diabetes and our findings held when controlling for other factors that may relate to decreased insulin sensitivity, namely age and waist circumference. These findings fit with earlier findings that fasting plasma insulin relates to low BRS after adjusting for body mass index, and the relationship between body mass index and BRS was mediated by fasting insulin levels [28]. Although the present study did not identify whether the changes in BRS were due to alterations in sympathetic or parasympathetic activity, it is important to note that BRS is an early and independent predictor of mortality risk [39,58]. As such, utilizing BRS as a marker of autonomic dysregulation is a particularly useful tool for identifying early autonomic dysfunction in populations that have not yet progressed to clinically significant levels of disease, such as type 2 diabetes. 

The present study has several limitations that should be noted. First, the present data are cross-sectional and therefore causality cannot be inferred. Future studies employing prospective designs or that manipulate circulating insulin levels will help to better understand the temporal sequence of events that contribute to the association between IR and BRS. It is likely that the relationships between IR, brain activity and BRS are complex and bi-directional, involving efferent and afferent dynamics. Understanding the temporal relationships between alterations of brain activity and peripheral physiology could potentially offer opportunities for early identification of early markers of dysregulation.

An additional limitation of the present study is the use of HOMA-IR as a measure of IR. Fasting insulin and glucose data (used to compute the HOMA-IR index) provide an estimate of IR that is suboptimal compared to more sensitive measures such as the hyperinsulinemic-euglycemic clamp. However, the HOMA-IR index is a widely-used measure that provides excellent correlation with more sensitive measures [59]. HOMA-IR values in the present study were comparable to previous studies examining ranges of IR in relatively healthy subjects, and 20% of the current sample had HOMA-IR values > 2.77 consistent with the top quintile of IR in population studies [60]. A significant portion of the sample was missing insulin data, almost entirely because of values that were below detectable thresholds in the context of the fasting protocol. Future studies employing more sensitive measures of IR, such as euglycemic clamps, will be necessary to fully characterize relationships between IR and BRS in individuals with low levels of IR.

The imaging protocol had several limitations that may have affected the present findings. The 5mm slice thickness used may not have been of adequate resolution to detect activity in certain regions associated with autonomic regulation and food intake, such as the hypothalamus. Also, the brainstem was outside the field of view. Future studies should employ specialized imaging methods for detecting brainstem activity [61,62] to more fully understand alterations in brainstem and forebrain components of the CAN that may be influenced by IR. Further, the amount of spatial smoothing required for arterial spin labeling studies may have also contributed to the lack of findings in other regions of the CAN. Future studies utilizing methods with increased spatial resolution may thus improve our understanding of more discrete regions that explain the relationship between IR and BRS. It will also be important for future work examining network connectivity dynamics within these brain systems to more fully understand how their activity and interactions with each other are altered in conjunction with IR. Finally, the present study did not examine structural brain characteristics. It is possible that volumetric and other structural factors may have contributed to partial-volume or other effects on rCBF. For example, indicators of metabolic dysregulation have been associated with volumetric and other structural changes in several recent studies[63]. Understanding how structural neural factors relate to functional neural alterations that covary with IR and rCBF is therefore an important future direction.

## Conclusions

In summary, the present study replicated the finding that IR inversely associates with BRS. A novel finding was the identification of regions in the CAN whose blood flow dynamics statistically mediated this relationship. These regions included the insula and perigenual cingulate cortices. These results suggest for the first time in humans that the central pathways linking insulin resistance to baroreflex sensitivity include forebrain regions involved in the homeostatic control of autonomic and cardiovascular activity. Understanding how insulin dysregulation relates to brain function may help characterize the pathways through which insulin resistance may lead to cardiovascular autonomic impairments and associated risk for adverse disease outcomes and premature mortality.

## Supporting Information

Figure S1 **Insulin resistance predicts baroreflex sensitivity (*b* = -0.16, p < 0.05).**
(DOCX)

Figure S2 **Insulin resistance is positively associated with resting cerebral blood flow.**
(DOCX)

Figure S3 **Resting cerebral blood flow is negatively associated with baroreflex sensitivity.**
(DOCX)

Table S1 **Bivariate correlations between variables of interest and covariates.**
(DOCX)

Table S2 **Regions of resting cerebral blood flow that associate with insulin resistance (**A**) and baroreflex sensitivity (**B**).**
(DOCX)
