# Supplementary figures and images for: Cerebral Blood Flow Links Insulin Resistance and Baroreflex Sensitivity
Source: PLoS One. 2013 Dec 16;8(12):e83288. doi: 10.1371/journal.pone.0083288 (PMC3865223; doi:10.1371/journal.pone.0083288)

Figure S1


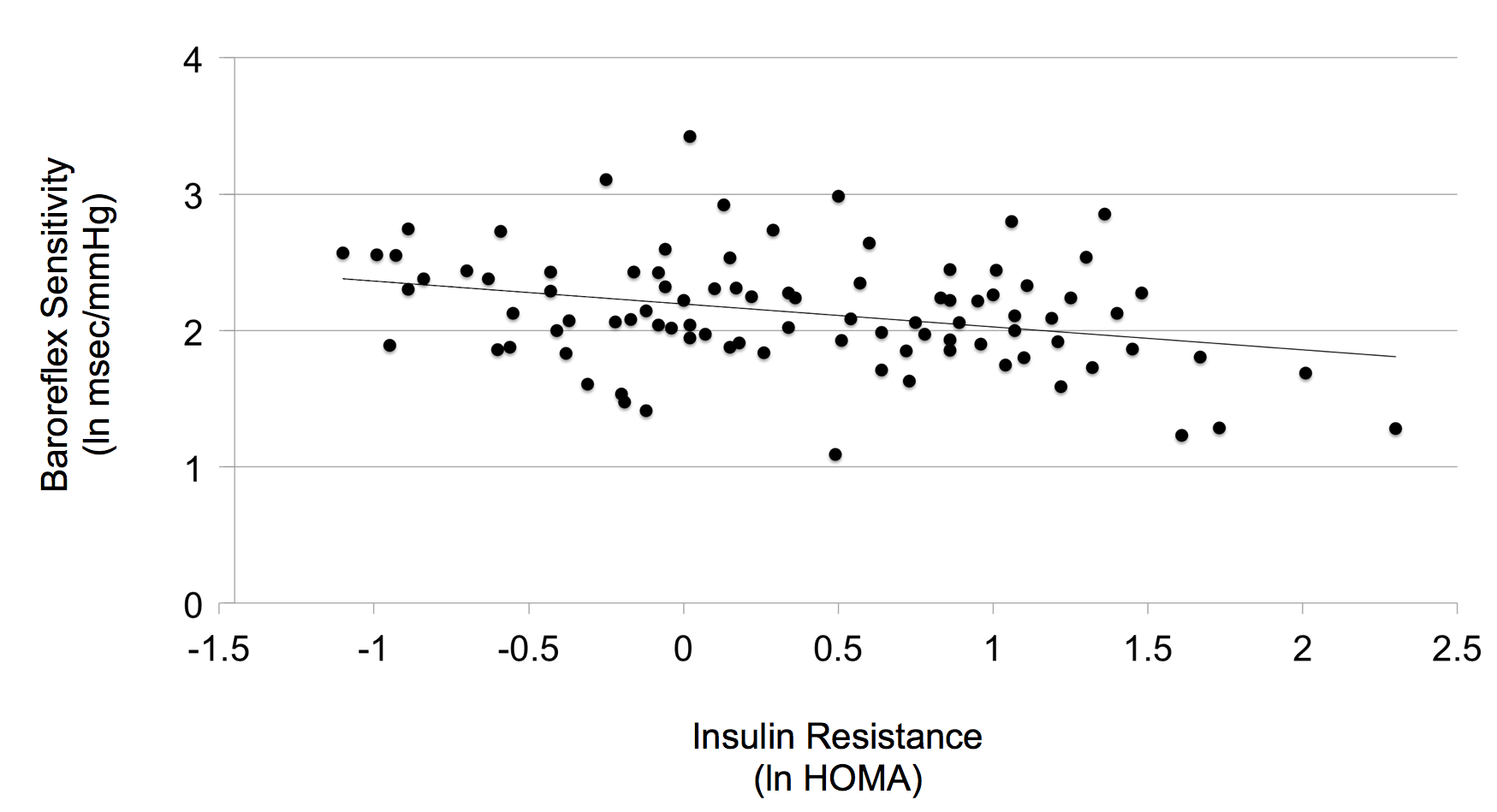

Supplement: Figure S1 — Insulin resistance predicts baroreflex sensitivity (b = -0.16, p < 0.05). (DOCX) [file pone.0083288.s001.docx]

Figure S2


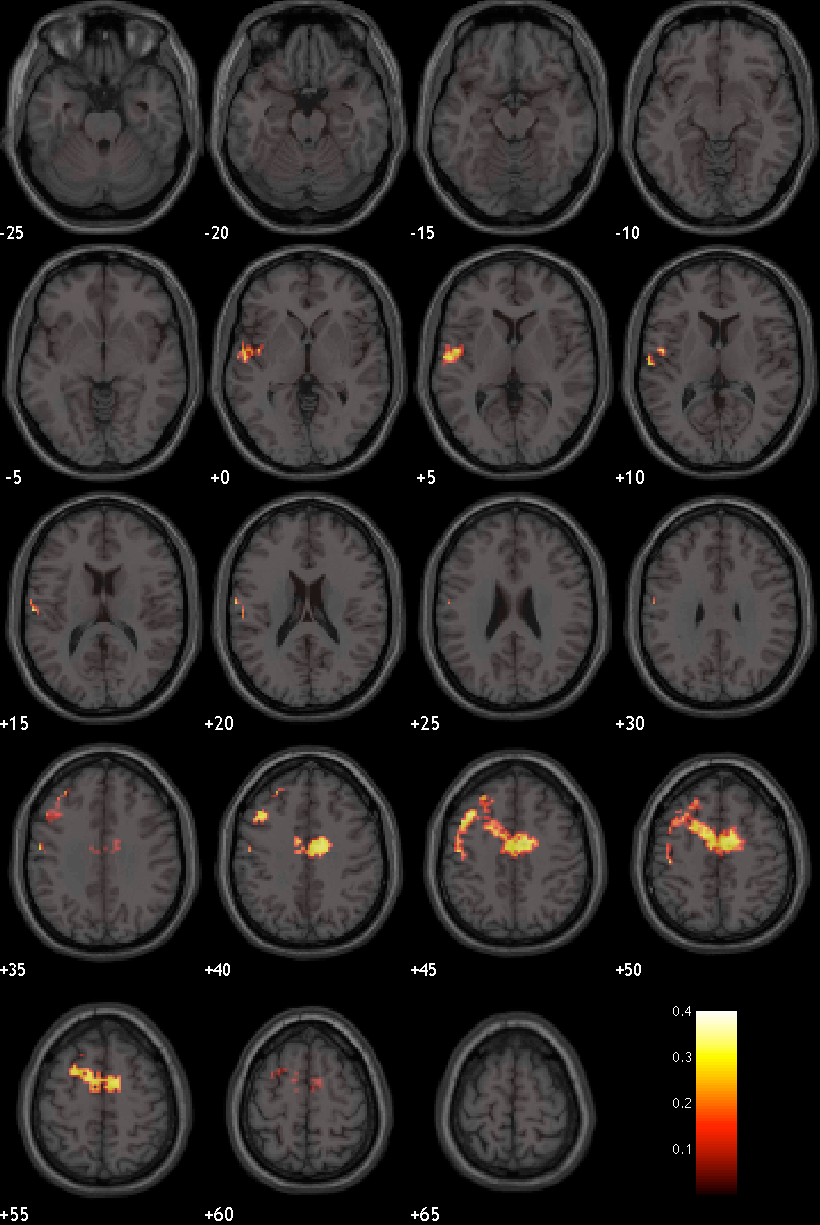

Supplement: Figure S2 — Insulin resistance is positively associated with resting cerebral blood flow. (DOCX) [file pone.0083288.s002.docx]

Figure S3


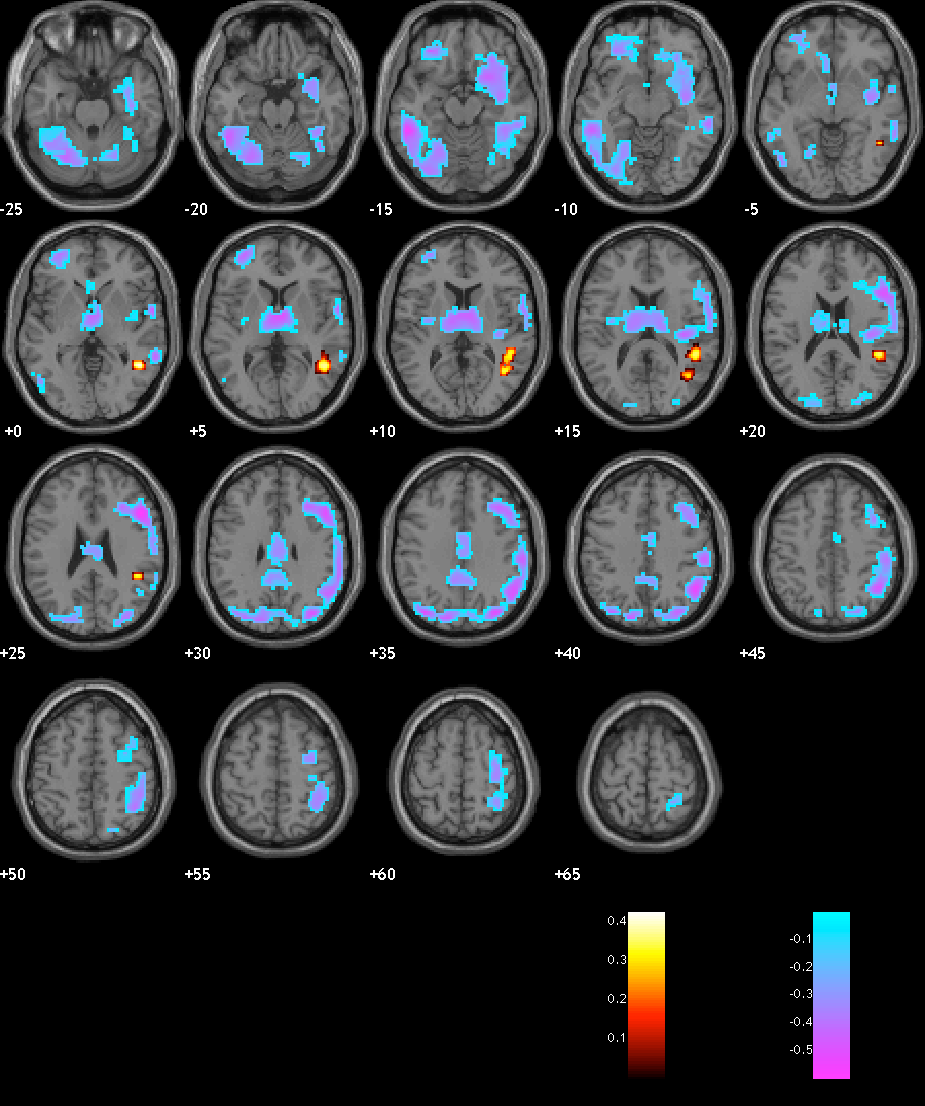

Supplement: Figure S3 — Resting cerebral blood flow is negatively associated with baroreflex sensitivity. (DOCX) [file pone.0083288.s003.docx]
