# Supplementary material for: Cerebral Blood Flow Links Insulin Resistance and Baroreflex Sensitivity
Source: PLoS One. 2013 Dec 16;8(12):e83288. doi: 10.1371/journal.pone.0083288 (PMC3865223; doi:10.1371/journal.pone.0083288)
Supplement: Table S1 — Bivariate correlations between variables of interest and covariates. (DOCX) [file pone.0083288.s004.docx]

Table S1

Bivariate correlations between variables of interest and covariates (N = 92).

|  | 1 | 2 | 3 | 4 |
| --- | --- | --- | --- | --- |
| 1. Baroreflex Sensitivity | - |  |  |  |
| 2. HOMA-IR | -.31** | - |  |  |
| 3. Waist Circumference | -.18 | .52** | - |  |
| 4. Age | -.22* | -.07 | .06 | - |
| 5. Systolic BP | -.29** | .35** | .63** | .11 |

HOMA-IR = Homeostatic Model Assessment of Insulin Resistance; BP = Blood Pressure.

** p* < .05

*** p* < .01
