# Supplementary material for: Cerebral Blood Flow Links Insulin Resistance and Baroreflex Sensitivity
Source: PLoS One. 2013 Dec 16;8(12):e83288. doi: 10.1371/journal.pone.0083288 (PMC3865223; doi:10.1371/journal.pone.0083288)
Supplement: Table S2 — Regions of resting cerebral blood flow that associate with insulin resistance (A) and baroreflex sensitivity (B). (DOCX) [file pone.0083288.s005.docx]

Table S2

Regions of resting cerebral blood flow (rCBF) that associate with insulin resistance (A) and baroreflex sensitivity (B). Next to each left (L) or right (R) side is an approximation of the brain region as well as the Brodmann Area if applicable. Montreal Neurological Institute (MNI) coordinates indicate the peak association between the variable of interest and rCBF for each cluster: x = right (+) to left (-), y = anterior (+) to posterior (-), z = superior (+) to inferior (-). Clusters are derived from a whole-brain analysis with an uncorrected height threshold of p < .005 and extent threshold of k = 109. Peak intensity values in the final column are for the voxel with the strongest association between rCBF and the variable of interest (insulin resistance or baroreflex sensitivity) and are derived from the probability maps generated from the BRAVO mediation toolbox. All regions displayed negative associations with baroreflex sensitivity, with the exception of one region marked by *.

| Side | Region | Brodmann Area | MNI Coordinates | | | Number of voxels | Peak intensity |
| --- | --- | --- | --- | --- | --- | --- | --- |
|  |  |  | x | y | z |  |  |
| (A) Regions associated with Insulin Resistance | |  |  |  |  |  |  |
| R, L | Midcingulate, Dorsolateral Prefrontal Cortex, Left Insula | 2, 3, 4, 6, 8, 9, 13, 22, 24, 31, 32, 42, 43 | -63 | -10 | 22 | 643 | 0.394 |
| (B) Regions associated with Baroreflex Sensitivity | |  |  |  |  |  |  |
| L | Posterior Temporal Lobe, Occipital Lobe, Cerebellum | 18, 19, 20, 21, 37 | -51 | -40 | -14 | 1163 | -0.569 |
| R | Insula, Inferior Frontal Gyrus, Amygdala, Parahippocampus | 11, 13, 20, 21, 22, 34, 38, 47 | 24 | 14 | -17 | 432 | -0.494 |
| R | Middle Temporal Gurus, Fusiform, Parahippocampus | 20, 21, 22, 37 | 63 | -43 | -2 | 197 | -0.471 |
| L | Orbitofrontal, Anterior Cingulate | 10, 11, 24, 25, 47 | -30 | 38 | -14 | 241 | -0.482 |
| R | Right Thalamus, Mid-Cingulate | 13, 23, 24, 31 | 6 | -4 | 7 | 647 | -0.522 |
| R* | Superior Temporal Gyrus, Inferior Parietal Lobule | 13, 19, 22, 39, 41 | 45 | -52 | 1 | 151* | 0.425 |
| R, L | Posterior Cingulate, Precuneus | 7, 31 | -3 | -43 | 34 | 134 | -0.368 |
| R | Dorsolateral Prefrontal Cortex, Cuneus, Insula, Operculum | 1, 2, 3, 4, 5, 6, 7, 8, 9, 18, 19, 21, 22, 31, 39, 40, 41, 42, 43, 46 | 48 | 20 | 25 | 1794 | -0.592 |

* region with positive peak intensity
